# Recent Lake Area Changes in Central Asia

**DOI:** 10.1038/s41598-019-52396-y

**Published:** 2019-11-07

**Authors:** Haijun Liu, Yaning Chen, Zhaoxia Ye, Yupeng Li, Qifei Zhang

**Affiliations:** 1 College of Resources and Environment Science/Key Laboratory of Oasis Ecology, Xinjiang University, Urumqi, 830046 China; 2 State Key Laboratory of Desert and Oasis Ecology, Xinjiang Institute of Ecology and Geography, Chinese Academy of Sciences, Urumqi, 830011 China

**Keywords:** Environmental sciences, Hydrology

## Abstract

Using Moderate Resolution Imaging Spectroradiometer (MODIS) 500 m spatial resolution global water product data, Least Squares Method (LSM) was applied to analyze changes in the area of 14 lakes in Central Asia from 2001 to 2016. Interannual changes in lake area, along with seasonal change trends and influencing factors, were studied for the months of April, July and September. The results showed that the total lakes area differed according to interannual variations and was largest in April and smallest in September, measuring −684.9 km^2^/a, −870.6 km^2^/a and −827.5 km^2^/a for April, July and September, respectively. The change rates for the total area of alpine lakes during the same three months were 31.1 km^2^/a, 29.8 km^2^/a and 30.6 km^2^/a, respectively, while for lakes situated on plains, the change rates were −716.1 km^2^/a, −900.5 km^2^/a, and −858 km^2^/a, respectively. Overall, plains lakes showed a declining trend and alpine lakes showed an expanding trend, the latter likely due to the warmer and wetter climate. Furthermore, there was a high correlation (r = 0.92) between area changes rate of all alpine lakes and the lakes basin supply coefficient, although there was low correlation (r = 0.43) between area changes rate of all alpine lakes area and glacier area/lake area. This indicates that lakes recharge via precipitation may be greater than lakes recharge via glacier meltwater. The shrinking of area changes for all plains lakes in the study region was attributable to climate change and human activities.

## Introduction

Lakes have a strong influence on both human beings and the ecological environment, providing water for local residents, developing fishery production, and playing an important role in agricultural irrigation^1–3^. Lake can also provide the necessary water conditions for vegetation in arid areas where precipitation is scarce and the ecosystem is fragile. As a geographical element in the arid region, lakes expansion or shrinkage are essential to agricultural development and the health of the plant and animal ecosystems inside and outside lake, such that land desertification and salinization in arid areas are severely impacted by lake area shrinkage^2,4^. Furthermore, the size of a lake exerts a regulating effect on the climate in the surrounding area^5^ by increasing or decreasing local air humidity and thus affecting precipitation in the lake basin. Therefore, it is critical not only for the development of socioeconomic status of countries in Central Asia but also for their ecological environmental protection to study variation of lakes area and analyze internal driving factors contributing to those changes.

**Figure 1:**
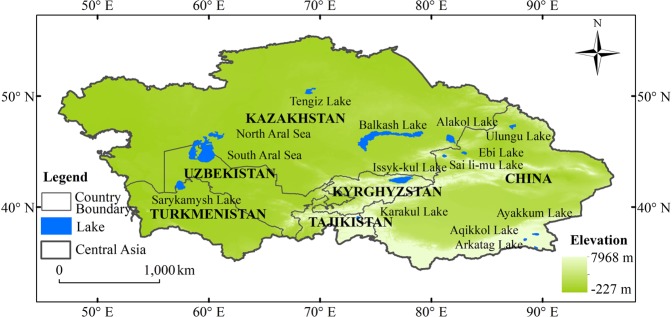
Location of the study area. (Generated by ArcGIS 10.2, URL: http://www.esri.com/sofware/arcgis/arcgis-for-desktop).

Central Asia is situated inland region from Eurasia (Fig. 1) and includes Kazakhstan, Tajikistan, Kyrgyzstan, Turkmenistan, Uzbekistan, and China’s Xin Jiang Province^6^. The region is characterized as arid and semi-arid, and the climate is mostly controlled by westerly air circulation. Annual precipitation in Central Asian countries varies greatly from region to region. In the windward (western) side of the Tianshan Mountains, precipitation amounts can top 2,000 mm annually, while in the desert side, precipitation levels are typically less than 100 mm^7^.Since the 1970 s, temperatures across Central Asia have shown an obvious rising trend of 0.368 ~ 0.428 °C/10 a^6,8^, which is higher than the current global average warming level. Overall, the vast region generally suffers from scarce water resources and a fragile ecological environment^9,10^. The largest water bodies of Central Asia are Aral Sea, and Issyk-kul Lake, Balkhash Lake (Table 1).

**Table 1:** 14 closed lakes greater than 200 km^2^ in Central Asia.

No	Lake Name^a^	Lon (°)	Lat (°)	Area (km^2^)	Elevation (m)	Lake type^b^
1	Issyk-kul Lake	77.43	42.49	6195.93	1601	Alpine lake
2	Sai li-mu Lake	81.16	44.60	460.42	2072	Alpine lake
3	Alakol Lake	81.67	46.15	2919.34	347	Alpine lake
4	Ayakkum Lake	89.43	37.56	616.34	3876	Alpine lake
5	Aqikkol Lake	88.41	37.08	354.71	4251	Alpine lake
6	Arkatag Lake	89.55	36.27	225.85	4713	Alpine lake
7	Karakul Lake	73.36	39.04	397.05	3915	Alpine lake
8	South-Aral Sea	59.71	44.91	23865.91	29	Plain lake
9	North-Aral Sea	60.95	46.34	2964.43	39	Plain lake
10	Sarygamysh Lake	57.45	41.89	3772.03	2	Plain lake
11	Tengiz Lake	68.89	50.41	1422.85	302	Plain lake
12	Balkhash Lake	74.60	46.39	16717.89	338	Plain lake
13	Ebi Lake	82.89	44.92	564.86	194	Plain lake
14	Ulungu Lake	87.29	47.28	854.89	478	Plain lake

^a^Information for lakes available at the HydroSHEDS website^40^ (http://www.hydrosheds.org/page/hydrolakes).

^b^Lake types are based on literature^41^.

In most cases, lake area change is the result of the combined action of climate change and human activities^2,3,11–16^. Climate change (e.g., changes in temperature and precipitation) directly impacts water cycle changes in a lake basin, while human activities (e.g., agricultural irrigation) can change the water cycle process in a lake system. In the context of global warming, the warming rate in Central Asia is higher than the average global warming rate^8^, which may promote the evaporation of the lake surface. The warming also speeds up the melting of glaciers and snow and brings the melting period forward.

In the plains region of Central Asia, changes in precipitation can cause changes in river runoff, which in turn can impact lake inflow and lake recharge and contribute to lake area expansion or shrinkage. Human activities such as irrigated agriculture consume water resources mostly in the form of evaporation and loss, which then directly affects lake area changes. Central Asia is a typical arid and semi-arid region in the inland temperate zone. Due to low levels of precipitation in this region, snow cover and glacier meltwater are important sources for lake water recharge. However, both snow and glacier cover are currently experiencing a shrinking trend due to changes of climatic factors.

Jing *et al*.^17^ extracted 12 lakes areas with a combined water index with MOD09A1 dataset in different seasons (April, July and September) in the year 2005–2015, but that only nearly 10-years long. Klein *et al*.^2^ used AVHRR and MODIS sensors to derive inland water bodies extents over a period from 1986 till 2012 for the region of Central Asia for the months of April, July and September. Tan *et al*.^3^ used MODIS NDVI data to extract areas of 24 lakes along the Silk Road (including some lakes of Central Asia) and analyze their spatial-temporal characteristics, but that only with annual mean lake area without seasonal changes. Li *et al*.^18^ also used Modis NDWI datasets to extract 9 lakes to analyze seasonal and inter-annual changes from 2001 to 2016, and the number of lakes was scarce. This paper used 14 lakes to analyze seasonal and inter-annual changes of 14 closed lakes with Modis product datasets.

Some studies have been conducted on changes in lake area, but most of the research was carried out within limited time frames^4,19^. Some images (eg. Landsat remote sensing images) have been used to obtain lake areas at a given moment for many times^16,20^, the lake area at a given moment indicates the area of the lake in a particular period, the lake area captured at a given point in time is insufficient to reflect inter-annual and annual variations because of the possible fluctuations of the lake area in the short term^3^. For example, one observation represents lake area for a year, thus lack of high temporal resolution (i.e., observing the lake area several times a year), and ignored annual seasonal changes affecting the lakes. Annual changes such as inundation and drought can cause a water body to fluctuate in area within a short time, resulting in significant impacts to the surrounding ecological environment. Currently, the data on lakes situated in arid regions do not fully reflect the variations in characteristics caused by annual changes to water surface area^3^, so the research which includes high temporal resolution is urgently required^21^. This paper studies the changes in lake water area in April, July and September in Central Asia and provides decision-making suggestions for water resource management and ecological environment maintenance for the impacted lakes.

In this paper, considering that the open lakes were highly regulated by reservoirs, we mainly considered closed lakes. The change of lakes water area in Central Asia was mainly affected by larger lakes, so this paper chose the main typical lakes (larger than 200 km^2^) in Central Asia, including 7 alpine lakes and 7 plain lakes. This paper contained all the great closed lakes in Central Asia except for the Caspian Sea, therefore, closed lakes larger than 200 km^2^ were selected as research objects.

## Results

### Temporal variation of lake area

**Figure 2:**
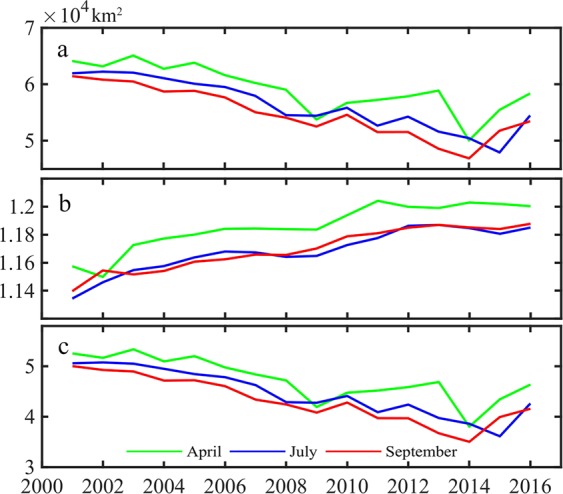
The total area change of all lakes, alpine lakes and plain lakes. (**a**) Total lakes area; (**b**) All alpine lakes area; (**c**) All plain lakes area). (Generated by Matlab 2018a, URL: http://cn.mathworks.com/products/matlab/).

For the years 2001 to 2016, the total area of the 14 lakes under study (Fig. 2a) was the largest in April, followed by July and September. The change rate for the total area of the lakes was −684.9 km^2^/a, P < 0.01, R^2^ = 0.63 in April, −870.6 km^2^/a, P < 0.05, R^2^ = 0.85 in July, and-827.5 km^2^/a, P < 0.01, R^2^ = 0.80 in September. The lake area decreased the fastest in July, followed by the area change rate in September. The area change rate in April was the lowest.

The change in total area of alpine lakes for the months of April, July and September (Fig. 2b) was largest in April, with little difference in the area during July and September. From 2001 to 2016, the change rate of the total area of the lake was 31.1 km^2^/a, P < 0.01, R^2^ = 0.84 in April, 30.6 km^2^/a, P < 0.05, R^2^ = 0.94 in July, and 29.8 km^2^/a, P < 0.01, R^2^ = 0.87 in September. The lake area increased the fastest in April, followed by the area change rate for July. The area change rate was the lowest in September.

The change in total area of lakes located in the Central Asian plains regions for the months of April, July and September (as shown in Fig. 2c) was the largest in April and the smallest in September. From 2001 to 2016, the change rates of the total area of the lakes in April, July and September were −716.1 km^2^/a, −900.5 km^2^/a, and −858 km^2^/a, with significance levels of P < 0.05 and R^2^ of 0.65, 0.86 and 0.81, respectively. The lake area decreased the fastest in July, followed by the area change rate for September. The area change rate was the lowest in April.

**Figure 3:**
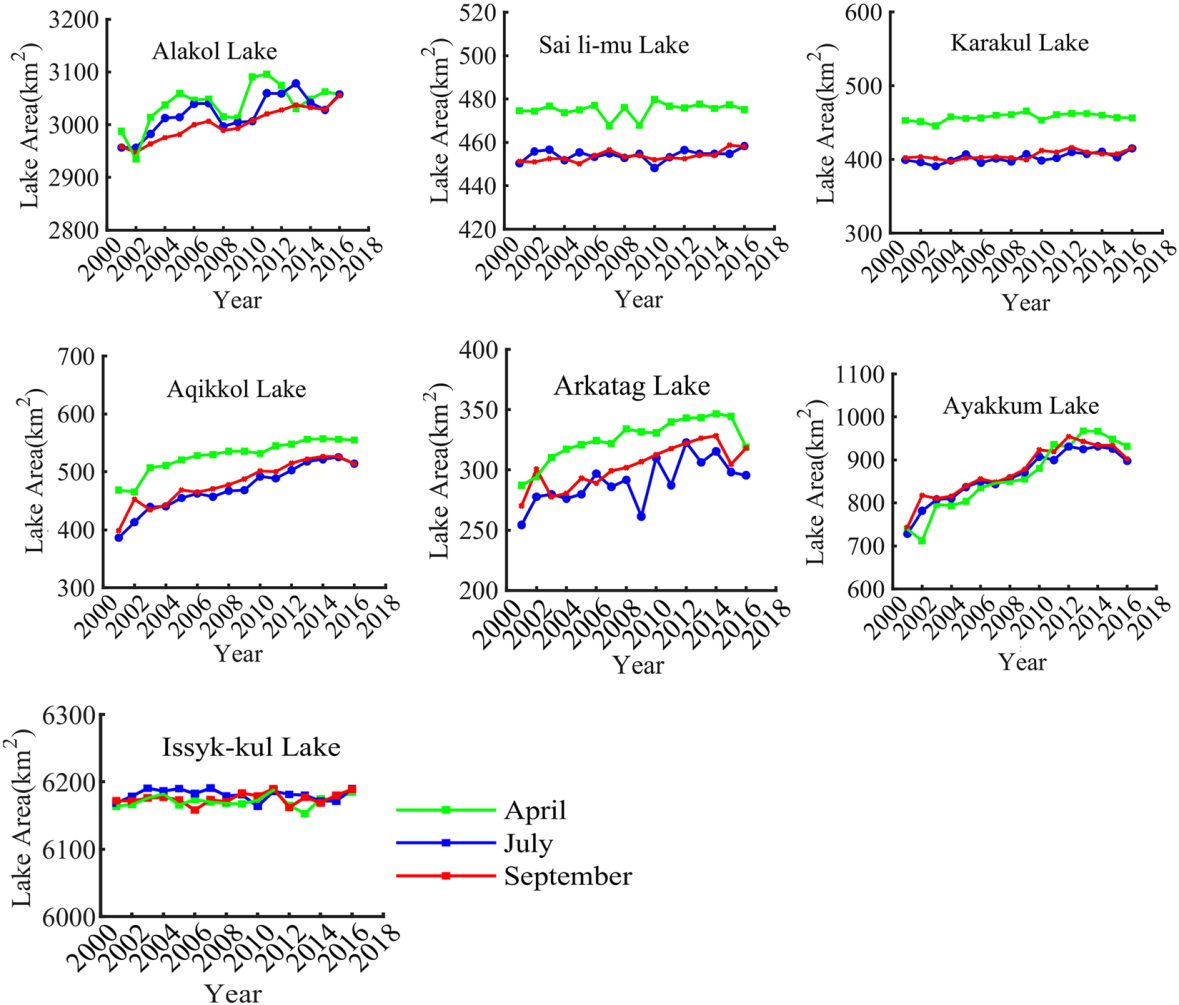
Interannual area variation of alpine closed lake in Central Asia in April, July and September from 2001 to 2016. (Generated by Matlab 2018a, URL: http://cn.mathworks.com/products/matlab/).

Figure 3 shows the average lake area for April, July and September as being the lake area for the entire year. From 2001 to 2016, the alpine lake area was either stable or expanding (Fig. 3, Table 2). For example, the area of Issyk-kul Lake was stable and did not pass the significance test level of P < 0.05. However, other lakes did pass the significance test level of P < 0.01. The annual change rates of Sai li-mu Lake and Karakul Lake were 0.21 km^2^/a and 0.81 km^2^/a, respectively. The change rates of Alakol Lake, Ayakkum Lake, Aqikkol Lake and Arkatag Lake were larger, ranging from 2.94 km^2^/a to 13.03 km^2^/a. According to Table 2, the seasonal variation rates of Aqikkol Lake, Arkatag Lake, Karakul Lake and Ayakkum Lake were 1.21, 1.27, 1.16 and 1.14, respectively, which were relatively large. On the other hand, the seasonal variation rates of Issyk-kul Lake, Sai li-mu Lake and Alakol Lake were 1.00, 1.07 and 1.03, respectively, indicating afairly small seasonal variation.

**Table 2:** Change in the trend lake in Central Asia from 2001 to 2016.

Lake Name	April (%)	July (%)	September (%)	Seasonality^a^ (max/min ratio (%))	Annual change rate (km^2^/a)
Issyk-kul Lake	0.73	0.72	0.67	1.00	0.48
Sai li-mu Lake	0.12	1.77	1.51	1.07	0.21**
Alakol Lake	2.33	3.42	3.29	1.03	5.77***
Ayakkum Lake	25.93	23.25	21.43	1.14	13.03***
Aqikkol Lake	18.48	32.94	28.77	1.21	7***
Arkatag Lake	11.12	16.14	17.77	1.27	2.94***
Karakul Lake	0.8	4.0	3.33	1.16	0.81***
South-Aral Sea	−27.79	−38.77	−42.13	1.99	−846.47***
North-Aral Sea	8.45	14.5	20	1.13	25.74***
Sarygamysh Lake	4.3	5	5.52	1.03	11.32***
Tengiz Lake	6.29	14.56	13.29	1.71	−16.5
Balkhash Lake	1.03	1.69	1.98	1.03	7.81
Ebi Lake	−1.66	10.95	17.63	1.45	−7.30*
Ulungu Lake	−0.15	1.13	0.4	1.08	0.5

^a^The method of seasonality for lakes area is based on literature^42^.

*Means significance level P < 0.05, **means significance level P < 0.01, ***means significance level P < 0.001.

**Figure 4:**
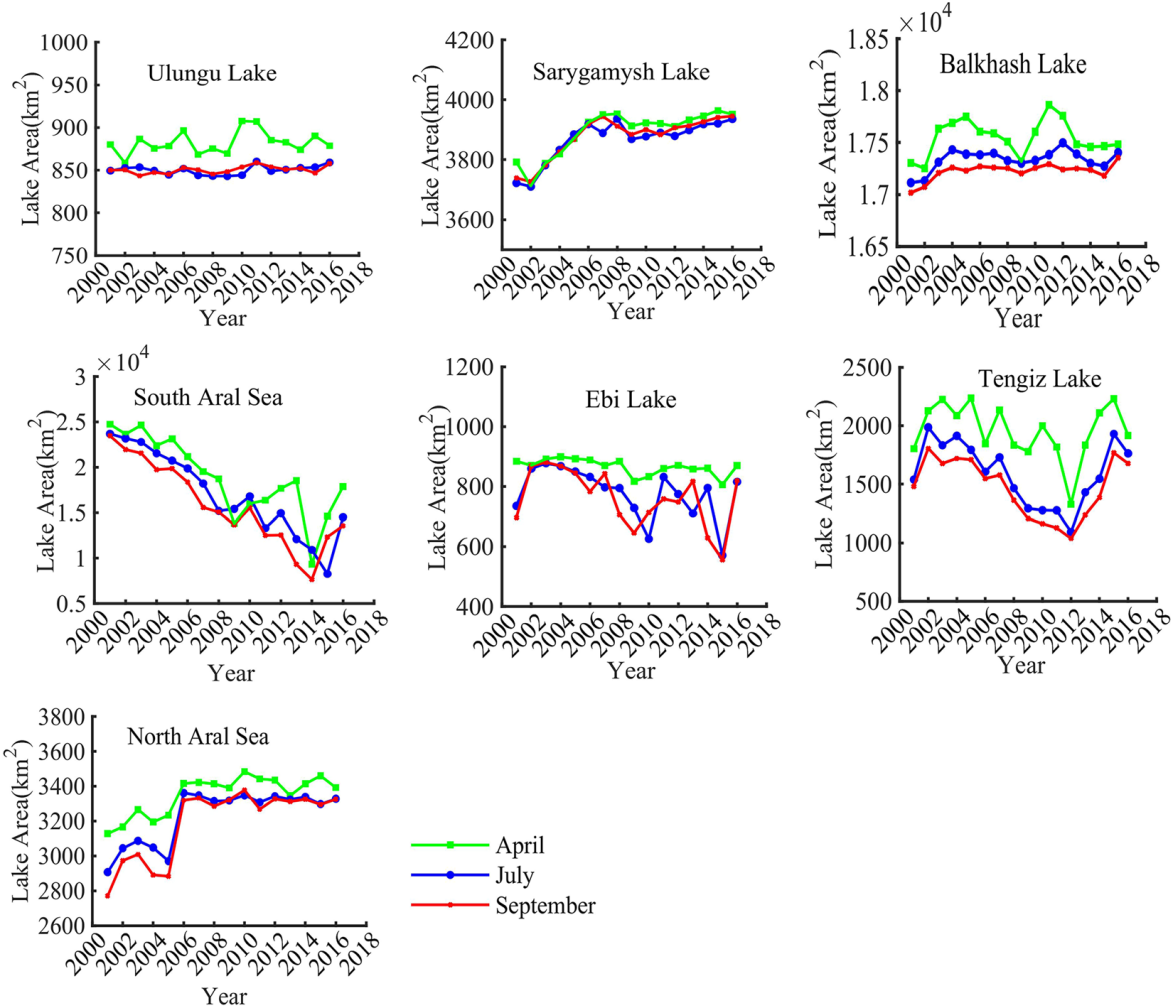
Interannual area variation of plain closed lake in Central Asia. in April, July and September from 2001 to 2016. (Generated by Matlab 2018a, URL: http://cn.mathworks.com/products/matlab/).

During the period under study, the area of plains lakes notably varied (Table 2 and Fig. 4). For example, the South Aral Sea, Ebi Lake and Tengiz Lake decreased, with the South Aral Sea and Ebi Lake passing the significance test of P < 0.001 and P < 0.05 and showing reduction rates of −846.47 km^2^/a and −7.30 km^2^/a, respectively. Conversely, the North Aral Sea, Sarygamysh Lake, Ulungu Lake and Balkhash Lake all exhibited an upward trend. Of these water bodies, the North Aral Sea and Sarygamysh Lake passed the significance test of P < 0.001 and showed increasing rates of 25.74 km^2^/a and 11.32 km^2^/a, respectively. According to Table 2, the seasonal variation rates of the South Aral Sea, Tengiz Lake and Ebi Lake were 1.99, 1.71 and 1.45, indicating that the seasonal variation rates of these lakes were relatively large, whereas the seasonal variation rates of Balkhash Lake, Sarygamysh Lake, Ulungu Lake and the North Aral Sea were 1.03, 1.03, 1.08 and 1.13, respectively, indicating little seasonal variation. Generally speaking, the seasonal variation rates of lakes on the Central Asian plains were larger than alpine lakes in the same region.

**Figure 5:**
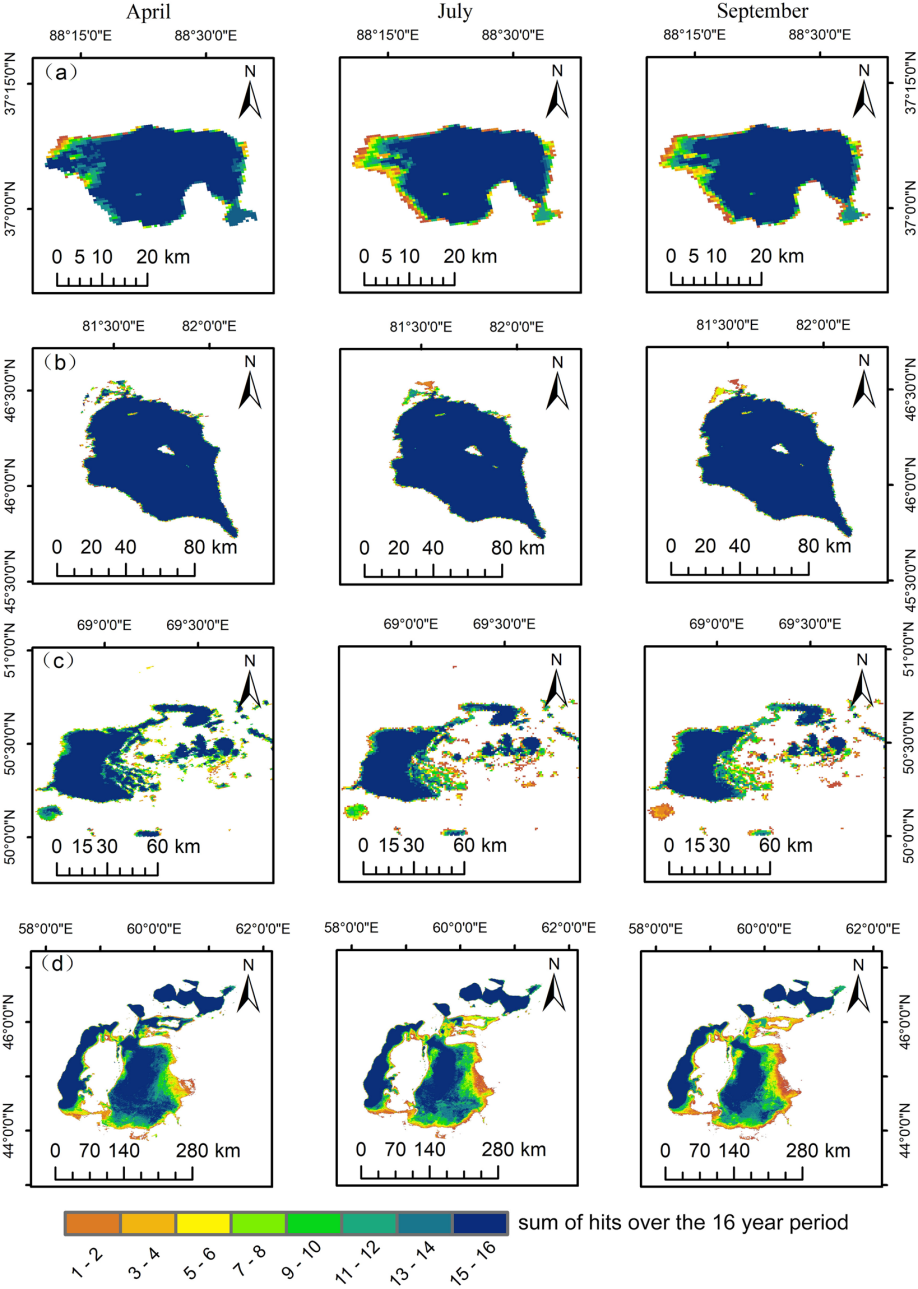
Lakes season dynamics of April, July and September from 2001 to 2016. (**a**) Aqqikol Lake, (**b**) Alakol Lake, (**c**) Tengiz Lake, and (**d**) South Aral Sea and North Aral Sea (Generated by ArcGIS 10.2, URL: http://www.esri.com/sofware/arcgis/arcgis-for-desktop).

The present study used Aqqikol Lake and Alakol Lake (Fig. 5a,b), along with Tengiz Lake and the North Aral Sea and South Aral Sea (Fig. 5c,d) as examples of seasonal variation of lakes. For April, July and September from 2001 to 2016, the seasonal variation of the alpine lakes Aqqikol Lake and Alakol Lake and North Aral Sea was not significant, whereas that of Tengzi Lake and South Aral Sea were quite significant. Specifically, the seasonal variation ratios were as follows: South Aral Sea 1.99; Tengiz Lake 1.71; North Aral Sea 1.13; Aqikkol Lake 1.21; and Alakol Lake 1.03. These ratios indicated that the seasonal variation map of lake dynamics was consistent with the seasonal variation ratio of the lakes (Table 2).

### Analysis of factors influencing lake area change

**Figure 6:**
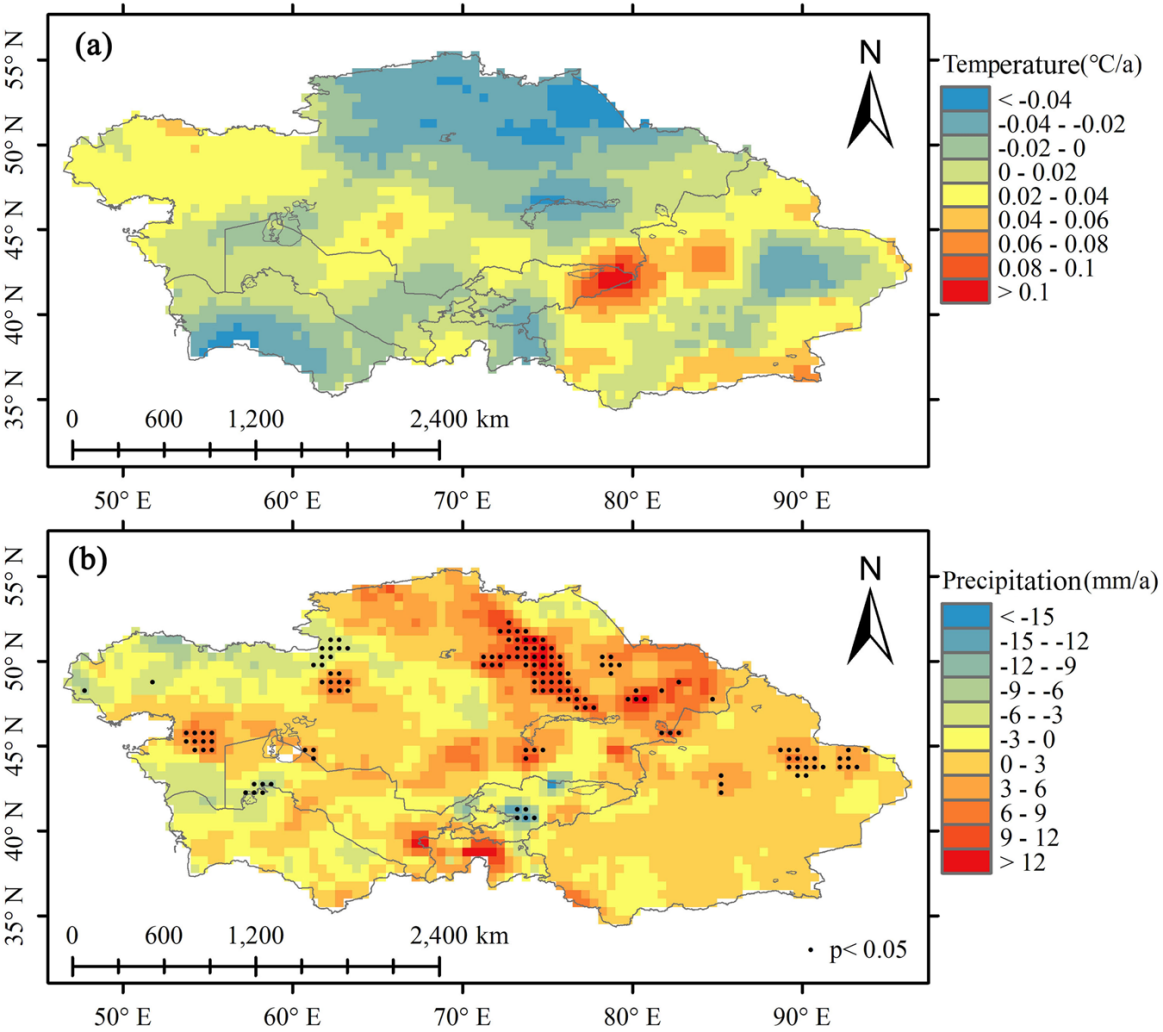
Trends of temperature and precipitation in Central Asia from 2001 to 2016. (**a**) Trend of Temperature change (**b**). Trend of precipitation change; Point presents the significance level p < 0.05). (Generated by ArcGIS 10.2, URL: http://www.esri.com/sofware/arcgis/arcgis-for-desktop).

Seven alpine lakes in the study area experienced an average warming rate of 0.053 °C/a, with the exception of Karakul Lake (Fig. 6a, Table 3). Precipitation also charted a general upward trend, (with the exception of Karakul Lake), with an average increase rate of 1.15 mm/a. The temperature rise in the lake basins not only accelerated the melting of snow and glaciers, but also lengthened the melting period, thus providing more water for the lakes. Additionally, the increase in rainfall supplied water directly through the lake surface as well as indirectly through runoff, which also played a role in the increase of lake area.

**Table 3:** Variation trends of temperature, precipitation and cropland in 14 lake basins.

Lake basin	Temperature (°C/a)	Precipitation (mm/a)	Cropland (km^2^/a)	Lake supply coefficients^a^ (basin/lake area ratio)	Glacier area/lake area^b^	Lake type^c^
Issyk-kul Lake	0.084	0.579	−13.03	3.54	0.08	A
Sai li-mu Lake	0.041	0.002	—	2.89	0.01	A
Alakol Lake	0.014	4.441	184.5*^d^	21.67	0.01	A
Ayakkum Lake	−0.031	−2.118	—	11.23	0.55	A
Aqikkol Lake	0.06	0.621	—	39.18	0.67	A
Arkatag Lake	0.053	0.332	—	37.39	0.28	A
Karakul Lake	0.065	0.925	—	21.44	0.97	A
South-Aral Sea	0.008	0.84	−150.1	26.08	—	P
North-Aral Sea	0.006	−0.243	−60.37	109.93	—	P
Sarygamysh Lake	0.004	−0.036	19.55*	19.15	—	P
Tengiz Lake	−0.019	2.813	−46.65	60.59	—	P
Balkhash Lake	0.008	1.943	−18.31	24.21	—	P
Ebi Lake	0.05	0.467	194*	91.53	—	P
Ulungu Lake	0.03	0.82	11.23*	46.46	—	P

^a^Lake supply coefficients is based on literature^43^.

^b^Glacier area/lake area is based on literature^43^.

^c^Lake type: “A” means alpine lake and “P” means plain lake in the lake type.

^d^*means significance level P < 0.05.

However, as indicated above, the precipitation and temperature in the Karakul Lake basin showed a downward trend which did not pass the significance test. As the precipitation was mainly concentrated in spring and summer^22^, the expansion of the lake may have been due to wintertime and springtime precipitation that melted in spring to form runoff as a source of water to recharge the lake. Karakul Lake was desiccative and strong evaporation due to temperature rise so runoff was mainly formed from glacier melt water in summer. Thus, lake area for spring was larger than summer and expanding.

**Figure 7:**
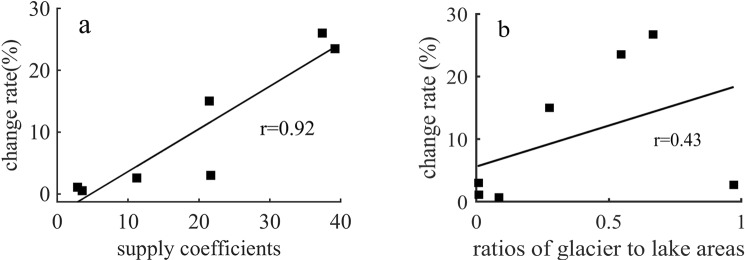
Relationship between alpine lake area change and supply coefficients, ratios of glacier to lake areas. (Generated by Matlab 2018a, URL: http://cn.mathworks.com/products/matlab/).

In the alpine lakes of Central Asia, the agricultural land area in the Issyk-kul basin showed a marked decrease, while the area of the Alakol Lake basin noticeably expanded (Table 3). However, the significance test of P < 0.001 indicated that changes in the area of Alakol Lake were mainly influenced by climate. By analyzing the relationship between lake area change rate and recharge coefficient, this study found a positive correlation, indicating that the relationship between lake area change and precipitation underwent significant changes^12,23^ (Fig. 7, Table 3). The correlation coefficient was r = 0.92. Furthermore, analysis of the glacier area/lake area (correlation coefficient r = 0.43) indicated that the ratio of glacier area/lake area was smaller than that of the lake recharge coefficient, pointing to precipitation being more obvious than glacier recharge (Fig. 7, Table 3).

North Aral Sea and South Aral Sea, along with the Sarygamysh Lake, Balkhash Lake, Ebi Lake, and Ulungu Lake basins, all experienced a warming trend (Fig. 6, Table 3), with an average warming rate of 0.018 C/a. During the same time period, temperatures in the Tengiz Lake basin showed a downward trend of −0.019 C/a. Meanwhile, precipitation in the South Aral Sea and Balkhash Lake, Ebi Lake and Ulungu Lake charted an increasing trend with an average increase rate of 1.38 mm/a, whereas the North Aral Sea and Sarygamysh Lake started a downward trend of −0.243 mm/a and −0.036 mm/a, respectively, with none of these water bodies passing the significance test. Agricultural land area in the South Aral Sea and North Aral Sea and Tengiz Lake and Balkhash Lake basins also exhibited a downward trend of −150.1 km^2^/a, −60.37 km^2^/a, −46.65 km^2^/a and −18.31 km^2^/a, respectively, again with none of these locations passing the significance test. In contrast, agricultural land in Sarygamysh Lake, Ebi Lake and Ulungu Lake showed a clear upward trend, with rising rates of 19.55 km^2^/a, 194 km^2^/a and 11.23 km^2^/a, respectively, which passed the significance test of P < 0.05. Under the dual climate conditions of rising temperature and rising precipitation, the plains lakes demonstrated a downward trend in area, indicating that they were affected by climate change and human activities. Human activities have different impacts on the utilization of water resources in the lake basin in different seasons, and it is difficult to obtain data on the amount of water consumed in each season. Therefore, we can only use cropland area to indirectly reflect the agricultural water consumption and analyze the reasons for yearly changes of lakes area.

## Discussion

In this paper, 14 Central Asian lakes with a combined area of more than 200 km^2^ were studied during the months of April, July and September to determine the seasonal variations in area. The freezing of the lakes in winter greatly affected their extraction accuracy^21^, so the winter season was not included in the study data. By comparing and analyzing the total area of lakes in April, July and September (representing changes in lake area during spring, summer and autumn), the study found a clear downward trend in the total area of the lakes, with alpine lakes showing an upward trend and plains lakes a downward trend.

It was concluded that the change of alpine lake area was generally increasing, while plain lake area was generally decreasing. The research conclusion was consistent with Tan *et al*.^3^ on the changes of lake area in Central Asia along the Silk Road, the plain lakes tended to shrink, such as Aral Sea, Ebi Lake and Sarygamysh Lake. The seasonal variation trend was similar as lake area obtained by Jing *et al*.^17^, such as Ebi Lake, Ulungu Lake and Ayakkum Lake. Bai *et al*.^24^ used Landsat images to study the changes in lake area of 9 inland lakes in Central Asia from 1975 to 2007, and found that the area of lakes in plain areas decreased significantly, while alpine lakes were relatively stable. These results were consistent with the research conclusions in this paper.

A dam between the North and South Aral seas was built in 2005 in the Berg Strait, completely controlling the water resources of the North Aral Sea. As a result, the recharge of the Syr Darya River into the North Aral Sea remained stable, meaning that the evaporation was in balance with lake precipitation and the runoff of the Syr Darya River into the lake^25^. However, the surface area remained stable only at certain times of the year. Snow melt water is an important water recharge source^26^, so the runoff in spring is higher than in summer and autumn. This finding was consistent with changes in lake area studied in this paper (Fig. 4).

The South Aral Sea experienced shrinking, followed by an increase. The springtime flooding of the Amu Darya River occurred frequently after early 2012, causing the surface of the South Aral Sea to rise^2^. At the same time, precipitation in the South Aral Sea region showed an increasing trend (Table 4), and rainy season from October to April^27^, leading to the South Aral Sea area being larger in spring than in summer or autumn due to frequent spring floods.

**Table 4:** Accuracy evaluation of lake area.

Lake Name	Date	Modis Water pixels	Landsat water pixels	User’s accuracy	Producer’s accuracy	Overall accuracy	Kappa coefficient
Ebi Lake	2002.10.30	3490	3405	0.90	0.92	0.94	0.87
Ayyakum Lake	2012.9.29	3785	3964	0.98	0.94	0.97	0.94
Ulungu Lake	2014.8.31	3454	3429	0.97	0.97	0.98	0.95

It is worth noting that the surface area of Sarygamysh Lake did not decrease but instead indicated an upward trend. The main reason for this seeming anomaly is that the farmland irrigation in the lower reaches of the Amu River did not flow into the Amu Darya River. Rather, Sarygamysh Lake was recharged by some of the water sources of the Amu Darya River^28^. Analysis of the relationship between lake area change and temperature and precipitation showed no obvious relationship among these factors, but there was a significant positive correlation between agricultural area expansion and lake area expansion (Table 4). The main reason was that the inflow of the external water source (the Amu Darya River) into Sarygamysh Lake caused expansion of the lake area. Thus, the changes which occurred to the lake’s surface area was primarily the result of human activities.

The main recharge sources of Tengiz Lake was inflow from snow-melt in Spring^29^. Thus area of Tengiz Lake for April was larger than July and September, and changed greatly. During the study’s time frame (2001–2016), the lake area decreased from 2001 to 2012, then enlarged from 2013 to 2015.The reasons may be climatic due to area reduction of cropland (−46.65 km^2^/a)in the Tengiz basin (Table 3). Precipitation in the Tengiz Lake basin from 2001 to 2016 was increasing indistinctively (2.813 mm/a), decrease of temperature (−0.019 °C/a) may cause drop in evaporation (Table 3), thus lake area change due to precipitation minus evaporation. The winter snow water equivalent in the Tengiz basin decreased from 2001 to 2012, and then began to increase subsequently^2,30^. Some small lakes changed with analogical trend in northern Kazakhstan due to increasing precipitation from 2013 to 2016^31^, these were consistent with our study.

Despite increasing trends for temperature and precipitation in the Ebi Lake basin, cultivated land area increased significantly and lake area decreased significantly. These changes were obviously influenced by human activities, which was consistent with the findings of the present study^32^. The development of irrigation agriculture in the upper reaches of the basin consumed water from rivers, directly affected the inflow of lakes, and reduced the area of lakes in the lower reaches. Plain lakes are mainly recharged by rivers, and lake surface area varies greatly according to the amount of river runoff. However, due to the lack of runoff data for lakes basin, this study had difficulty quantitatively analyzing the impact of runoff for lakes.

Precipitation and temperature in Ayakkum Lake, Aqikkol Lake, Arkatag Lake and Sai-limu Lake basins also showed an upward trend^33^. According to the findings of a recent study, Alakol and Issyk-kul lakes were either stable or expanding in area due to rising temperatures in the nearby mountain region^34^. For Karakul Lake, which is situated in the Pamir Plateau, precipitation and temperature were slightly decreasing, thus inhibiting evaporation. Moreover, because Karakul Lake is surrounded by mountains, it is difficult for the wet vapor flow of west wind circulation to enter the basin^35^, which left the lake area in a stable state.

Lake ice was identified as part of lake area for April in this paper, the present study noted that lakes area were larger than that reported in the existing literature due to different reflectivity for ice and water^2,17^. There may be also some uncertainty with large water bodies, as the spatial resolution of MODIS is 500 m each day, which is relatively low.

## Conclusion

The present work studied area changes occurring from 2001 to 2016 in 14 typical lakes in Central Asia during the months of April, July and September. Using daily 500 m resolution water product data, the interannual and seasonal variation characteristics of lakes were analyzed. Overall, the total area of the 14 lakes under study showed a significant decreasing trend. Specifically, the change rates for lake area in April, July and September were −684.9 km^2^/a, −870.6 km^2^/a and −827.5 km^2^/a, respectively. The total area of lakes situated in plains regions showed a significant decreasing trend during the months of April, July and September, with change rates of −716.1 km^2^/a, −900.5 km^2^/a and −858 km^2^/a, respectively. However, the total area of lakes situated in alpine regions showed a significant increasing trend, with change rates of 31.1 km^2^/a, 29.8 km^2^/a and 30.6 km^2^/a for the same three months, respectively.

The study findings also showed that the area change rate of alpine lakes was less than that of plains lakes. The seasonal variation rates of lakes in the plains region of Central Asia ranged from 1.03 to 1.99, with seasonal variation rates for the South Aral Sea, Tengiz Lake and Ebi Lake being1.99, 1.72 and 1.45, respectively. The seasonal variation of alpine lakes was smaller than that of plains lakes, ranging from 1 to 1.27. The seasonal variation rates of Issyk-kul Lake, Sai-limu Lake and Alakol Lake (in the Tianshan Mountains) were slightly less, ranging from 1 to 1.07, while the rates for Aqikkum Lake, Aqikkol Lake, Arkatag Lake and Karakul Lake (in the Kunlun Mountains and Pamir Plateau)were between 1.14 and 1.27.

Analysis of the factors influencing alpine lake area changes points to the warm and humid climate likely being the main cause for the expansion. Hence, seasonal variations in lake area differed according to recharge source and the proportion of the components. For instance, alpine lake area changes were highly positively correlated with the lake basin recharge coefficient (r = 0.92), whereas the changes showed only a slight correlation with glacier area/lake area (r = 0.43) (Fig. 7). The recharge of precipitation to lakes may be greater than glaciers.

For the plains lakes, the shrinkage of surface area was primarily the result of climate change and human activities. Furthermore, even though the area of agricultural land in the South Aral Sea basin declined, the decrease in the area of the lake was due to a portion of the runoff from the Amu Darya River recharging the Sarygamysh Lake and subsequently increasing the lake area. The North Aral Sea situation differed substantially from that of the South Aral Sea, as the truncation of surface water sources caused by the Berg Strait dam resulted in the basin basically achieving water balance, with only a slight increase in area. In the same region, Ebi Lake was directly affected by human activities of agricultural irrigation water consumption and the decrease of water inflow into the lake. In contrast, Balkhash and Ulungu lakes saw an increase in their lake areas due to the warm and humid climate surrounding them. Finally, the Tengiz Lake basin underwent a slight cooling and humidifying change in climate, which may be related to an increase inlake area after year of 2013. Through the analysis of the causes of lake changes, this paper provided suggestions for water resources management in lake basins.

## Methods

### Annual change rate of lake area

Least Squares Method (LSM)^3^ was used to calculate annual change rate of lake area, as follows:

1 $${\rm{y}}=\frac{{\rm{n}}\times {\sum }_{{\rm{i}}=1}^{{\rm{n}}}{\rm{i}}\times {{\rm{x}}}_{{\rm{i}}}-({\sum }_{{\rm{i}}=1}^{{\rm{n}}}{\rm{i}})({\sum }_{{\rm{i}}=1}^{{\rm{n}}}{{\rm{x}}}_{{\rm{i}}})}{{\rm{n}}\times {\sum }_{{\rm{i}}=1}^{{\rm{n}}}{{\rm{i}}}^{2}-{({\sum }_{{\rm{i}}=1}^{{\rm{n}}}{\rm{i}})}^{2}}$$

where n represents the number of years, x_i_ represents the annual average, and y represents the slope of the linear regression equation. A positive rate of change indicates an upward trend, whereas a negative rate of change indicates a downward trend.

### Trend changes of temperature and precipitation

Mann-Kendall test was used to calculate the climate change rate and significance level^36^. The result (P < 0.05) indicates that the trend passed the significance test.

### Extraction of lakes area

The downloaded MODIS product data were sinusoidal projection, which required projection transformation to Albers equal area with Python, with projection parameters for the central longitude 105°E, two standard parallel latitudes 25°N and 47°N, respectively, and a resampling resolution of 500 m. The vector mask file of 14 lakes was used as a buffer to obtain an area about three times the size of each lake. Water files were then extracted through the buffer zone, with the extraction process batch processed in Python. After extraction, attribute values of the.tif data with code of 61 (water) and 62 (lake ice) was assigned to 1, then.tif data were binarized to remove small water bodies outside the lakes, and monthly mean values were obtained through daily area statistics.

### Data sources

Precipitation and temperature data for the study area were sourced from GPCC V8 (https://www.dwd.de/EN/ourservices/gpcc/gpcc.html) and GHCN_CAMS (https://www.esrl.noaa.gov/psd/data/gridded/data.ghcncams.html), both with spatial resolution of 0.5°and monthly temporal resolution.

Daily water body product data for the lake from 2001 to 2016, with spatial resolution of 500 m (http://data.ess.tsinghua.edu.cn/modis_500_2001_2016_waterbody.html), which was generated from MOD09GA.006 with the processing algorithm^21^. The MODIS tiles corresponding to lakes in this paper are h22v03, h22v04, h23v05, h23v04 and h25v05.The monthly data for lake area were calculated by means of the daily data of lake water in April, July and September, and include 14 closed lakes (7 alpine and 7 plains) with an area larger than 200 km^2^ (Table 1). We considered that alpine lakes were mainly located in alpine regions with small watershed areas and deep lakes^12,37^. Lake boundary and basin boundaries shapefiles were sourced from USGSHydroSHEDS (https://hydrosheds.cr.usgs.gov/datadownload.php?). Randolph Glacier Inventory6.0(RGI6.0) was used for the glacier lake basin (http://www.glims.org/RGI/rgi60_dl.html). MCD12Q1.006 from 2001 to 2016 (https://ladsweb.modaps.eosdis.nasa.gov/search/)with a resolution of 500 m was also employed. Landsat TM/Oil 30 m lakes data^38^ for validation were download from http://www.sciencedb.cn/dataSet/handle/621.

### Precision evaluation of Lake water

In order to evaluate the data accuracy, we compared the results from the Landsat image explanation and MODIS product data of the Ayyakum Lake, Ebi Lake and Ulungu Lake. Landsat lake files were resampled to 500 m spatial resolution as Modis, keeping the same projection with Modis Albers projection, Modis raster data were compared with Landsat data pixel by pixel. The comparison results were shown in Table 4. The overall accuracy were all above 0.90, and the Kappa coefficient was above 0.90. Only Ebi Lake Kappa coefficient was 0.87, which may be a mixed pixel with great uncertainty in classification^39^.
